# Successful pedicled vertical rectus abdominis myocutaneous flap reconstruction with negative-pressure wound therapy for deep sternal wound infection: a case report and comprehensive review

**DOI:** 10.3389/fsurg.2023.1268555

**Published:** 2023-11-06

**Authors:** Dong Yun Lee, SuRak Eo, SooA Lim, Jung Soo Yoon

**Affiliations:** Department of Plastic and Reconstructive Surgery, DongGuk University Medical Center, Seoul, Republic of Korea

**Keywords:** vertical rectus abdominis myocutaneous (VRAM) flap, deep sternal wound infection (DSWI), negative-pressure wound therapy (NPWT), cardiac injury, case report

## Abstract

**Introduction:**

Deep sternal wound infection (DSWI) is a serious complication that may occur after median sternotomy, with potentially devastating consequences. By reporting our case and analyzing the existing literature, this article aimed to provide a thorough understanding of the role of negative-pressure wound therapy (NPWT) and the importance of flap choice in managing DSWI accompanied by severe heart injury and high hemodynamic risk.

**Case description:**

A 60-year-old woman with severe aortic stenosis, aortic valve regurgitation, and heart failure underwent redo sternotomy, which resulted in an intraoperative right ventricle injury. She required extracorporeal membrane oxygenation support because of low blood pressure and subsequently developed complications, including surgical site hematoma, wound dehiscence, and fat necrosis. She was referred for wound closure, where a significant 10 × 20-cm soft tissue defect in the anterior chest wall was observed. A pedicled vertical rectus abdominis myocutaneous flap addressed the soft tissue defect. The wound showed remarkable improvement at the 8-month follow-up visit.

**Conclusions:**

DSWI management is a complex and multifaceted challenge. NPWT, when combined with appropriate surgical strategies, including wound debridement and flap selection, may promote successful wound healing. This case report highlights the successful management of a complex DSWI using a multidisciplinary approach, including debridement, appropriate antibiotic therapy, and free-flap reconstruction, which resulted in favorable outcomes.

## Introduction

Deep sternal wound infection (DSWI) is a serious complication that may occur after median sternotomy, with potentially devastating consequences. The incidence of DSWI remains significant, ranging from 0.25% to 5% (1), with varying rates of postoperative mortality depending on the isolated bacterial strain, typically between 3% and 13% (1–5). Researchers have explored innovative modalities such as negative-pressure wound therapy (NPWT) to improve treatment outcomes and promote successful wound healing. NPWT, introduced in the late 1990s, has emerged as a promising treatment approach for DSWI (6). Its ability to stabilize the sternum, promote granulation tissue formation, and facilitate wound perfusion is attributed to several mechanisms. This method aims to optimize wound healing and ultimately achieve delayed primary closure, thereby reducing the risk of complications. In addition to NPWT, selecting an appropriate flap for wound coverage is critical in successful DSWI management (5–8). The choice of the flap is influenced by several factors, including the size of the wound and the patient's specific characteristics. Local flaps, such as the pectoralis major flap, transverse rectus abdominis myocutaneous (TRAM)/vertical rectus abdominis myocutaneous (VRAM) flap, omentum majus flap, and latissimus dorsi (LD) flap, have been used for sternal wound reconstruction (8–10). These flaps offer vascularized tissue that may promote healing and cover large defects. However, the decision regarding flap choice is not solely based on the wound size. The location and extent of sternal loss, previous interventions, and the presence of comorbidities also influence the selection process. When determining the most appropriate option for each case, it is crucial to consider factors such as flap vascularity, tissue thickness, and donor site morbidity.

By reporting our case and analyzing the existing literature, this article aimed to provide a thorough understanding of the role of NPWT and the importance of flap choice in the management of DSWI. The insights gained from this investigation may aid clinicians in optimizing treatment strategies, improving patient outcomes, and minimizing the risk of sternal wound complications.

## Case description

A 60-year-old woman with hyperlipidemia presented to the emergency room with severe aortic stenosis, moderate aortic valve regurgitation, and heart failure. The patient underwent open aortic valve repair (valvuloplasty) to treat aortic stenosis. Four days later, an evacuation was necessary because of a postoperative hematoma, which resulted in sternal instability and DSWI. Intravenous piperacillin and tazobactam were administered for two weeks, and a wound swab culture and a tissue culture revealed methicillin-sensitive *Staphylococcus aureus* (MSSA). During the initial period of DSWI, intravenous cefazolin was administered, and the patient exhibited a positive response.

After three weeks of multiple debridements and NPWT application changed twice a week for the open wound on the anterior chest, three consecutive wound swab cultures and tissue cultures identified no bacterial growth. As no signs of wound infection were noted, a repeat sternotomy was subsequently performed in the cardiothoracic surgery department to achieve sternal fixation and wound closure, ensuring sternal stability. Unfortunately, this resulted in a right ventricle injury due to severe adhesion and fibrosis in the retrosternal space. Owing to the patient's rapidly declining blood pressure, extracorporeal membrane oxygenation (ECMO) was initiated with vasopressor administration, and the patient was immediately moved to the intensive care unit (ICU). Daily dressing using an open-wound approach was applied to the defect with an exposed pericardium, and delayed flap reconstruction was planned.

**Figure 1:**
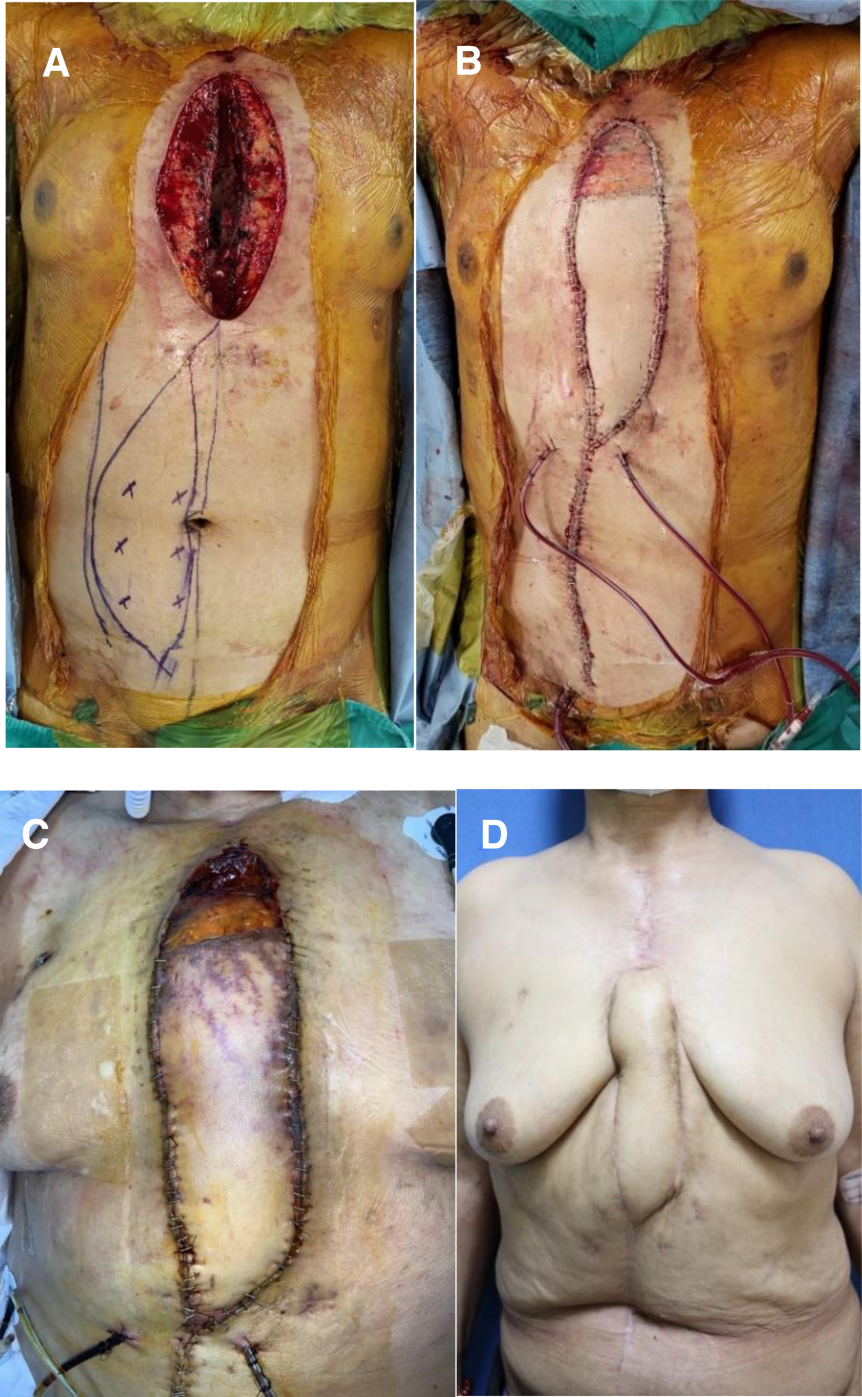
Pedicled vertical rectus abdominis myocutaneous (VRAM) flap reconstruction of a 60-year-old female with a deep sternal wound infection. (**A**) Preoperative view. (**B**) Intraoperative view, immediately after inset of a right pedicled VRAM flap for coverage of the anterior chest defect. (**C**) Partial necrosis of the flap was observed on the ninth postoperative day. (**D**) Postoperative 8 mo view (anterior view); the wound recovered without any surgical complications.

The patient was referred to our department for soft tissue defect coverage three days after the second sternotomy and ventricle injury. Upon examination, a significant 10 × 20-cm defect in the anterior chest wall was discovered, extending from the jugular notch to the xiphoid process (Figure 1A). Necrotic tissue was visible, and the heart was exposed. Careful curettage and debridement were performed to avoid damage to the heart, and the margins of the nonviable tissue were trimmed. Approximately one-fourth of the upper sternum remained intact, providing stability as it was connected to both clavicles and rib bones. In contrast, for the lower portion, the viable sternum accounted for about 50% of its preoperative width. To address the soft tissue and bony defect, a pedicled VRAM flap was used for coverage (Figure 1B). ECMO was decannulated on the same day, and the patient's vital signs stabilized.

Following flap reconstruction, the patient experienced partial flap congestion due to vascular insufficiency, leading to partial necrosis in the distal one-quarter of the flap on the ninth postoperative day (Figure 1C). Subsequently, debridement of necrotic soft tissue was performed, and wound closure was facilitated with the assistance of NPWT. On the twelfth postoperative day, methicillin-resistant *Staphylococcus aureus* (MRSA) was identified in the tip culture of the Hemovac inserted underneath the flap. There was no direct clinical evidence of infection. However, with concerns regarding flap site infection or the recurrence of DSWI, intravenous vancomycin was promptly administered for two weeks. This treatment continued until tissue cultures revealed negative bacterial growth at the open wound site following partial flap necrosis. It is essential to clarify that the vancomycin administration was not aimed at treating mediastinitis (DSWI), as the presence of MRSA was not considered the causative bacteria of the mediastinitis. Vacuum-assisted closure (VAC) was performed twice a week in the operating room, accompanied by copious irrigation with betadine solution. After one week, the secondary wound bed was covered with healthy granulation tissue without signs of infection, and primary closure was performed without tension on the bilateral skin. Fortunately, complete wound healing was achieved in the third postoperative month after the initial flap surgery. During the 8-month follow-up period, the patient showed excellent recovery progression and was healthy (Figure 1D).

## Discussion

Mediastinitis and DSWI are severe and potentially fatal complications that may occur after a median sternotomy. Preoperative risk factors associated with mediastinitis and DSWI include diabetes mellitus, obesity, advanced age, heart failure, left ventricular dysfunction, smoking, female sex, underlying chronic kidney disease, peripheral vascular disease, prolonged preoperative hospital stay, and emergency surgery (1, 11). Prolonged procedure duration and perfusion time are considered intraoperative risk factors (9). Postoperative risk factors include respiratory failure and an extended ICU stay. In our case, the patient was an elderly female with heart failure with multiple risk factors for prolonged hospital stay, particularly in the ICU.

Management of mediastinitis and DSWI involves implementing appropriate antibiotic therapy and surgical strategies (1–3). Prompt identification of the causative microorganism and determination of antibiotic susceptibility are crucial after diagnosis of sternal wound infection. Studies have indicated that *Staphylococcus aureus* (*S. aureus*) is the most commonly implicated pathogen (12). However, there has been a notable increase in the incidence of DSWI caused by slow-growing pathogens such as coagulase-negative *staphylococci* (12). Compared with MSSA mediastinitis, MRSA mediastinitis is associated with a significantly higher mortality rate, up to an 11-fold increase (1). Vancomycin is typically the preferred choice for treating most MRSA cases (12), followed by oral therapy options such as rifampicin, quinolones, and co-trimoxazole. Fortunately, in the present case, the patient was diagnosed with MSSA mediastinitis, and intravenous cefazolin was initiated during the early stages of DSWI development. The patient responded favorably to the cefazolin treatment. Subsequently, after flap reconstruction, partial flap necrosis occurred, and MRSA was identified in the tip culture of the Hemovac drain. While there were no direct clinical signs of infection, concerns regarding the potential recurrence of infection led to the administration of intravenous vancomycin until tissue cultures confirmed the absence of bacterial growth.

Surgical strategies for managing mediastinitis and DSWI include various approaches such as revision with open dressing, primary closure, closed irrigation, NPWT, and reconstruction using vascularized soft tissue flaps (11). Although the optimal surgical approach for treating sternal infections remains a topic of debate, there is a consensus that wound debridement is necessary in all cases (3). Regardless of the treatment method, it is crucial to ensure sternal stability before delayed primary closure (5). In cases of DSWI in which extensive debridement has been performed and the sternum is unstable, a steel plate may be used for fixation prior to delayed wound closure. Soft tissue flap transposition is often required to address sternal defects resulting from multiple treatment interventions or to fill a sternal wound directly after debridement. However, in the present case, fixation was not feasible because of a prior sternectomy and recurrent wound infection (2–4).

The size of sternal wounds may vary significantly, and some patients may present with wounds larger than 400 cm^2^, requiring specialized coverage based on a specific concept. The prevailing approach involves utilizing local flaps, such as the pectoralis major flap, TRAM/VRAM flap, omentum majus flap, or, occasionally, the LD flap (5). The selection of an appropriate flap depends on the size and location of the sternal defect. Unilateral myocutaneous pectoralis major flaps are suitable for small defects, unilateral pedicled pectoralis major flaps for medium-sized defects, and pedicled LD flaps for larger defects exceeding 12 cm (3). In cases of significant inferior sternal loss, a rectus abdominis flap may be required in combination with a pectoralis flap, and the choice of flap depends on the specific characteristics of the patient's DSWI defect. It is important to note that sternal reconstruction using muscle flaps may present challenges, including chronic pain and/or sternal instability, reported in more than 40% of cases, and long-term muscle weakness (5–8).

In our case, the pectoralis major muscle was not possible because of the large defect size, including skin defects, after debridement. Owing to concerns regarding the patient's hemodynamic instability with lateral positional changes, using a pedicled LD flap for defect coverage was not feasible. Instead, a pedicled VRAM myocutaneous flap was selected to cover the entire sternal defect, including the upper, middle, and lower portions. Preoperative abdomen CT revealed reliable rectus muscle flap vascularity. A two-team approach for flap elevation was possible during debridement by the thoracic and cardiovascular surgery teams in the same operative field. This decision ensured optimal tissue coverage while minimizing the risks associated with the patient's condition.

Introduced in the late 1990s, NPWT is a relatively new treatment approach offering several sternal wound management benefits. It is employed following debridement and prior to primary closure or flap reconstruction to promote healthy granulation, enhance vascularity, and control infection, thereby creating an optimal environment for successful reconstruction. NPWT involves a specific technique in cases of DSWI (1–5). The specific technique involves the following method: After debridement of the sternal wound, multiple layers of paraffin gauze can be placed at the bottom to protect the pericardium and the heart from potential damage, and the sternum might not be firmly fixed using internal devices. Subsequently, a polyurethane foam is subsequently tailored to fit the wound and covered with a sterile drape. A tube for transmitting pressure is attached through a hole in the drape, enabling the application of negative pressure. This closed NPWT wound dressing has been shown to enhance postoperative wound healing and minimize complications such as seroma, hematoma, and infection (6). In our specific case, NPWT served as a valuable salvage procedure for addressing the partial failure of flap reconstruction, effectively reducing the need for another flap surgery and facilitating complete wound healing through VAC.

In conclusion, the management of DSWI is a complex and multifaceted challenge. This report highlights a case in which flap reconstruction was successfully performed on an unstable patient with complications following aortic valve replacement for aortic stenosis and heart failure. NPWT, as a salvage procedure to address flap compromise, specifically partial necrosis, plays a crucial role in achieving successful surgical outcomes. We report this case because efficient chest wall reconstruction was performed by optimizing the surgical treatment and NPWT. With this case report, we aim to contribute more to the literature.

## Data Availability

The original contributions presented in the study are included in the article/Supplementary Material, further inquiries can be directed to the corresponding author.

## References

[1] Abu-Omar Y Kocher GJ Bosco P Barbero C Waller D Gudbjartsson T European Association for cardio-thoracic surgery expert consensus statement on the prevention and management of mediastinitis. Eur J Cardiothorac Surg. (2017) 51(1):10–29. 10.1093/ejcts/ezw326 28077503

[2] Weinand C Xu W Perbix W Theodorou P Lefering R Spilker G. Deep sternal osteomyelitis: an algorithm for reconstruction based on wound width. J Plast Surg Hand Surg. (2013) 47(5):355–62. 10.3109/2000656X.2013.769441 23710791

[3] Schiraldi L Jabbour G Centofanti P Giordano S Abdelnour E Gonzalez M Deep sternal wound infections: evidence for prevention, treatment, and reconstructive surgery. Arch Plast Surg. (2019) 46(4):291–302. 10.5999/aps.2018.01151 31336416 PMC6657195

[4] Kass JL Lakha S Levin MA Joseph T Lin HM Genden EM Intraoperative hypotension and flap loss in free tissue transfer surgery of the head and neck. Head Neck. (2018) 40(11):2334–9. 10.1002/hed.25190 30230116

[5] Kaul P. Sternal reconstruction after post-sternotomy mediastinitis. J Cardiothorac Surg. (2017) 12(1):94. 10.1186/s13019-017-0656-7 29096673 PMC5667468

[6] Lalezari S Lee CJ Borovikova AA Banyard DA Paydar KZ Wirth GA Deconstructing negative pressure wound therapy. Int Wound J. (2017) 14(4):649–57. 10.1111/iwj.12658 27681204 PMC7949947

[7] Kitano D Takahashi H Nomura T Okada K Terashi H Sakakibara S. A new clinical classification and reconstructive strategy for post-sternotomy surgical site infection. Regen Ther. (2022) 21:519–26. 10.1016/j.reth.2022.10.007 36382133 PMC9634152

[8] Levy AS Ascherman JA. Sternal wound reconstruction made simple. Plast Reconstr Surg Glob Open. (2019) 7(11):e2488. 10.1097/GOX.0000000000002488 31942289 PMC6908337

[9] Liu H Liu J Wu Y Ma Y Zhou M Xue Y Analysis of the risk factors for free flap necrosis in soft tissue reconstruction of the lower limbs. Orthop Surg. (2023) 15(6):1534–40. 10.1111/os.13727 37092532 PMC10235166

[10] Wyckman A Abdelrahman I Steinvall I Zdolsek J Granfeldt H Sjöberg F Reconstruction of sternal defects after sternotomy with postoperative osteomyelitis, using a unilateral pectoralis major advancement muscle flap. Sci Rep. (2020) 10(1):8380. 10.1038/s41598-020-65398-y 32433505 PMC7239941

[11] Pagotto VPF Gallafrio ST Carneiro IC Gemperli R Jatene FB. Treatment and chest reconstruction for mediastinitis following sternotomy for cardiac surgery at the heart institute of the university of São Paulo medical school. Braz J Cardiovasc Surg. (2021) 36(4):565–70. 10.21470/1678-9741-2020-0117 33577255 PMC8522307

[12] Ma JG An JX. Deep sternal wound infection after cardiac surgery: a comparison of three different wound infection types and an analysis of antibiotic resistance. J Thorac Dis. (2018) 10(1):377–87. 10.21037/jtd.2017.12.109 29600070 PMC5863177

